# Respiratory Effects of Benzodiazepine in Patients with Advanced-Stage Cancer Receiving Opioid Analgesics: A Prospective Observational Study

**DOI:** 10.1089/pmr.2024.0021

**Published:** 2024-09-10

**Authors:** Akiko Yoshimura, Natsuko Nozaki-Taguchi, Dai Suganuma, Yoshihiko Sakashita, Masami Fujisato, Shiroh Isono

**Affiliations:** ^1^ Division of Palliative Care, Chiba Cancer Center, Chiba, Japan.; ^2^ Department of Anesthesiology, Graduate School of Medicine, Chiba University, Chiba, Japan.; ^3^ Palliative Care Center, Chiba Cancer Center, Chiba, Japan.

**Keywords:** additive effect, advanced-stage cancer, benzodiazepine, opioid, palliative care, respiratory depression

## Abstract

**Background:**

Despite the risk of respiratory depression, benzodiazepines are often prescribed to patients receiving palliative care owing to their efficacy in symptom control. Opioids, which also cause respiratory depression, are often administered to patients with advanced-stage cancer. However, the additive effect of the two drugs has not been systematically analyzed.

**Objective:**

This prospective observational study aimed to determine the respiratory effects of coadministration of benzodiazepines and opioids in terminally ill patients with cancer.

**Methods:**

The respiratory variables (primary endpoint) and activity index (ACI) (secondary endpoint) of 24 patients were assessed using a continuous noncontact, nonrestraining vital sign monitor placed under the legs of the bed.

**Results:**

The respiratory rate (RR) changed from 12.0  ±  3.9/min to 10.3  ±  3.3/min (*n* = 24, *p* = 0.0005) following administration of the first dose of benzodiazepine in addition to regular opioid treatment, indicating no difference (*p* > 0.83) from the decrease in the RR observed on the previous day at the same time (12.1  ±  3.3/min to 10.3  ±  3.4/min). No increase in apnea–hypopnea frequency and respiratory irregularity or no decrease in respiratory size was observed. The ACI showed a significant decrease following the administration of benzodiazepine, suggesting remission of the symptoms. The effect of five repeated doses of benzodiazepines in nine patients showed no significant change in the respiratory variables compared with the first dose.

**Conclusion:**

Addition of single or consecutive benzodiazepine-type drugs at clinically useful dose in patients receiving palliative care for cancer with opioid analgesics, readily exposed to respiratory depression, was observed with a decreased RR similar to the decrease observed during sleep with opioid alone.

## Introduction

Patients with advanced-stage cancer often report disturbances in sleep patterns that correlate with the symptoms of anxiety/depression.^1^ The appropriate management of these symptoms plays a crucial role in maintaining the quality of life of these patients. Benzodiazepine-type hypnotics and anxiolytics are prescribed to 58%–75% of patients in Western hospices^2^ because of their efficacy in managing insomnia, depression, and anxiety, as well as the symptoms of dyspnea, helplessness, nausea, and pain. The short-term use of benzodiazepine is associated with the risk of respiratory depression. Patients with advanced-stage cancer often receive opioids for pain relief, which also increases the risk of respiratory depression. Daytime evaluation of respiration has shown that symptom-relieving doses of opioids are safe^3^; however, respiratory depression is often observed at night during sleep when nursing staff or caregivers are scarce. Polysomnograms acquired during sleep have shown frequent episodes of central obstructive apnea in patients receiving opioids.^4^ Data acquired over a 48-hour period using nonrestraining respiratory monitor data revealed nighttime opioid dose-dependent respiratory rate (RR) depression in patients with terminal cancer. Moreover, RR irregularity and ataxic respiratory abnormalities were also observed, especially in female patients.^5^ Few systematic studies have evaluated the effects and side effects of the administration of benzodiazepine to terminally ill patients with cancer receiving opioids, and the results of the studies have been inconsistent.^2,6^

Determining the respiratory effects of benzodiazepines in patients with advanced-stage cancer receiving opioids via 24-hour monitoring of the respiratory status would be beneficial. Therefore, this prospective observational study aimed to assess the respiratory patterns of these patients after coadministration of benzodiazepines and opioids using a continuous noncontact, nonrestraining vital sign monitor placed under the bed.

## Methods

### Ethical setting

The Ethics Committee of the Chiba Cancer Center approved the protocol for this prospective observational study (IRB R03-003), along with confirmation that the research was completed in accordance with the Declaration of Helsinki as revised in 2013. The study was registered with the UMIN Clinical Trial Registry (UMIN000046363; principal investigator: Y.S.; date of registration: October 20, 2021).

### Sample size calculation and study population

It was hypothesized that nonintravenous administration of benzodiazepine would induce a significant decrease in the RR of palliative care patients receiving opioids. Thus, the primary endpoint was set as the changes in the RR after the administration of benzodiazepine. A previous study by our group showed that the RR at night was 12.2  ±  3.5/min in patients in the palliative care ward at high risk of experiencing respiratory events.^5^ A decrease of ≥3 breaths/min in the RR was expected after the administration of benzodiazepine in the present study, given that bradypnea of ≤10 breaths/min is clinically harmful. The sample size was estimated to be 13 cases when α, 1-β, standard deviation (SD), and effect size were 0.05, 0.8, 3.5, and 3, respectively. Forty patients were enrolled in this study owing to the possibility of dropout from patients not receiving opioids or benzodiazepines. Approximately 50% of patients receive opioids and concurrent benzodiazepines during hospitalization at our department.

Among the patients with cancer admitted to the palliative care unit at our institution, those who met the following criteria were recruited in this study: terminal stage of cancer requiring palliative treatment, non head and neck cancer, no requirement for tracheostomy, and ability to provide written informed consent. Written informed consent was obtained from each patient admitted to the palliative care unit after explaining the risks and purpose of the study.

The demographic data of the patients were obtained at the time of enrollment. Patient age, sex, and cancer diagnosis were recorded. The Eastern Cooperative Oncology Group Performance Status (0–4) and Karnofsky Performance Status (0%–100%) scores were used to evaluate the performance status of the patients.^7^ The Palliative Prognostic Index^8^ and Palliative Prognostic Score were used to evaluate the prognosis of the patients.^9^ The Japanese version of the Edmonton Symptom Assessment System (ESAS-r) was used to record the physical symptoms.^10^

Opioids, benzodiazepines, and other medications were administered for the management of symptoms, such as insomnia, depression, anxiety, dyspnea, restlessness, nausea, and pain, within the usual clinical practice. The regular administration of opioids (oral sustained-release formulations or continuous infusion) and administration of the first dose of benzodiazepines were confirmed from the medical chart. Data were analyzed for the initial administration of benzodiazepines in all patients. The effects of repeated dosing were determined for those who lived 3–14 days after the first dose and had received more than five repeated doses.

### Bed sensor vital sign monitoring system

Continuous monitoring with the Bed Sensor System (BSS) (MinebeaMitsumi, Nagano, Japan) commenced at the same time as receiving consent and continued until discharge. The precise specifications and mechanism of the BSS have been described in previous reports.^5,11^ Four load cell sensors were placed under the wheels of the bed, and continuous signals were sent to the data logger for data processing and analysis. The four load cells independently measured the total weight on the sensors by detecting small movements, such as centroid shift due to heart beats, respiration, and slight body movements on the bed to total bed leave. The measurable vital signs included RR, apnea–hypopnea frequency, respiratory tidal weight (TW) change, and heart rate. The stability indices of RR (RRSI) and TW (TWSI) were calculated after determining RR. RRSI is defined as the percentage change in respiratory intervals calculated for every breath and averaged per hour. TW is defined as the gram change in weight owing to respiration that parallels the tidal volume.^12^ TWSI is defined as the percentage change in weight changes owing to respiration. Apnea–hypopnea frequency is defined as the number of episodes per hour of more than 10-second duration of 50% reduction of tidal weight or complete cessation of respiration. The magnitude of the weight shift on the sensors was calculated as the acceleration (g・s^−2^) of the movements and termed activity index (ACI). All signals and variables were processed at a sampling rate of 10 Hz within the data logger and stored on a personal computer connected to the data logger for analyses. The output of continuous BSS monitoring provided a summary of the hourly median or average values, depending on the vital signs monitored. The hour during which benzodiazepine was administered was referred to as T0. The preceding hour was referred to as T-1. The first to sixth hours following the administration of benzodiazepine were referred to as T1–T6.

### Statistical analysis

Normally distributed continuous variables are presented as mean and SD. Non-normally distributed continuous variables are presented as median with range (min–max). The ACI and RR during the 6 hours of observation were compared using Friedman Repeated Measures Analysis of Variance on Ranks. Multiple comparisons with the Control Group (Dunnett’s Method) were performed. The apnea–hypopnea frequency, RRSI, TW, and TWSI were compared between T0 and T when the RR was at the lowest within 6 hours using the *t*-test or rank-sum test, where appropriate. The effect of repeated dosing was analyzed using the Friedman Repeated Measures Analysis of Variance on Ranks.

## Results

Forty Asian patients receiving palliative care between January 2022 and September 2022 were enrolled in this study. Sixteen patients were excluded from the analysis as they did not require coadministration of opioids and benzodiazepines for symptom management. Thus, the BSS data of 24 patients were included in the final analysis. Of these, nine patients received repeated benzodiazepine doses and survived for 3–14 days.

### Patient characteristics

The patients included in this study were in the terminal stage of cancer, with a median Karnofsky Performance Scale of 40 and a short predicted prognosis (Table 1).

**Table 1.** Patient Characteristics (*n* = 24)

Age, year	71.3 ± 10.6
Sex (male, female)	(13,11)
Location of cancer, % (number)	
Digestive organs	54 (13)
Respiratory/intrathoracic	17 (4)
Breast	4 (1)
Urinary tract	13 (3)
Male genital organ (include prostate)	4 (1)
Female genital organs	4 (1)
Unknown/Other	4 (1)
ECOGPS, median (min–max)	3 (1–4)
KPS, median (min–max)	40 (40–70)
PPI, median (min–max)	5.5 (3.5–9.5)
PPS, median (min–max)	40 (20–70)
ESAS-r, median (min–max)	
Pain	5 (0–8)
Tiredness	6 (0–8)
Drowsiness	4 (0–9)
Nausea	0 (0–7)
Appetite	7 (0–10)
Shortness of breath	0 (0–10)
Depression	2 (0–10)
Anxiety	3 (0–10)
Well-being	6 (0–9)
Chief complaint at the time of admission, % (number)	
Pain	46 (11)
Nausea	21 (5)
Bloating	17 (4)
Dyspnea	17 (4)
Morphine milligram equivalents, median (min–max)	
(mg/kg/day)	0.85 (0.3–14.1)
(mg/day)	39 (15–480)

Values are presented as mean ± SD, % (number), and median (min–max].

ECOG-PS, Eastern Cooperative Oncology Group Performance Scale; ESAS-r, Japanese version of Edmonton Symptom Assessment System; KPS, Karnofsky Performance Scale; PPI, Palliative Prognostic Index; PPS, Palliative Prognostic Score.

The assessment of physical and psychological symptom control using the ESAS-r revealed high scores for pain, tiredness, drowsiness, and loss of appetite.

Benzodiazepines were administered as suppository drugs (*n* = 16; 67%) or oral medication (*n* = 8; 33%). The dose of each drug and number of patients were as follows: rectal diazepam (4 mg; *n* = 15), oral diazepam (1 mg; *n* = 1), oral lorazepam (0.5 mg; *n* = 3), oral brotizolam (0.25 mg; *n* = 2), oral alprazolam (0.4 mg; *n* = 1), rectal bromazepam (3 mg; *n* = 1), and oral clonazepam (0.5 mg; *n* = 1). Benzodiazepines were administered to manage insomnia (40%), nausea (17%), anxiety (13%), dyspnea (10%), pain (7%), stomach distention (7%), helplessness (3%), and psychiatric disturbances (3%). The choice of drug and dosage were determined according to the clinical routine followed at our hospice. The dosage of benzodiazepines was calculated as diazepam equivalent according to the psychotropic dose equivalence,^13^ whereas that of opioids was calculated as morphine equivalent dose. The median morphine equivalent dose of opioids administered was 39 mg/day (range: 15–480 mg).

### Changes in ACI after the administration of benzodiazepines

**FIG. 1.**
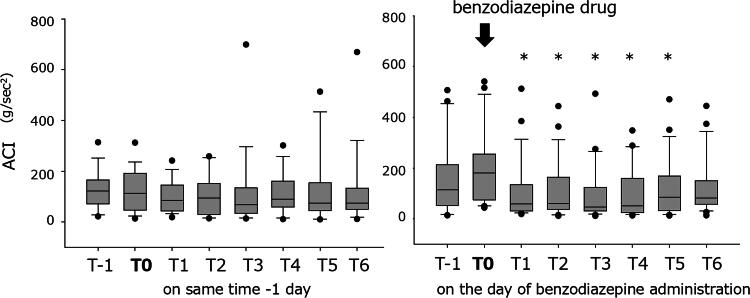
The median (interquartile range) of the activity index (ACI; g・s^−2^) and acceleration of movement on the bed before and after the administration of benzodiazepine (right graph: *n* = 24) showed a significant decrease in the ACI 5 hours after the administration of benzodiazepine (*p* < 0.001; Friedman Repeated Measures Analysis of Variance on Ranks, post-hoc test: Dunnett’s Method). The hour including the time taken to administer benzodiazepine is termed as T0. The preceding hour is termed as T1. The first to sixth hour after the administration of benzodiazepine is termed as T1–T6. The ACI of the day before (left graph: *n* = 18) shows constant level of ACI at the same level as benzodiazepine treated.

The ACI is expressed as an acceleration of movements in the bed (g・s^−2^), which serves as a good indicator of sleep and decreases during the nighttime.^14,15^ Benzodiazepines were administered during the daytime to five patients for reasons other than insomnia. However, the ACI (Fig. 1) showed a significant decrease compared with that at T0 after the administration of benzodiazepines for 5 hours (*p* < 0.001, Friedman Repeated Measures Analysis of Variance on Ranks, post-hoc test: Dunnett’s Method), indicating possible remission of the symptoms. A comparison of the ACI after the administration of benzodiazepines with that at the same time on the previous day (if applicable) revealed a similar level of ACI. Thus, the patients experienced some symptoms at T0, and an increase in the activity on bed was observed that ceased after the administration of benzodiazepines.

### Changes in respiration (primary endpoint)

**FIG. 2.**
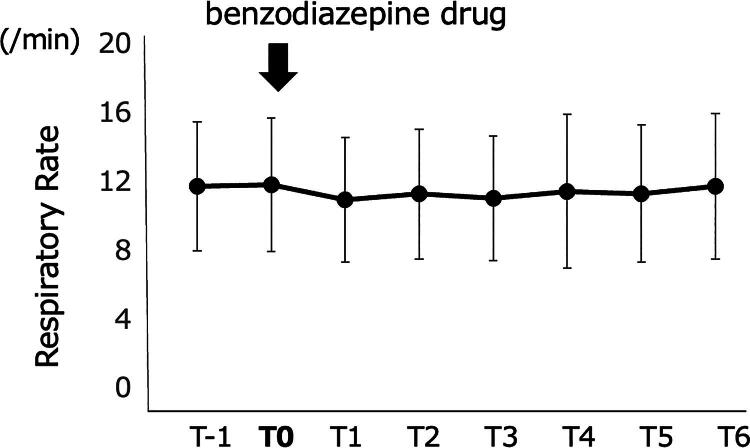
The hourly average respiratory rate (mean ± standard deviation: *n* = 24) before and after the administration of benzodiazepine shows constant level. The hour, including the time taken to administer benzodiazepine, is termed as T0 and the preceding hour is termed as T1. The first to sixth hour after the administration of benzodiazepine is termed as T1–T6.

The RR shown as an hourly average after the administration of benzodiazepines showed no change during 6 hours of continuous measurement (Fig. 2). It was hypothesized that a decrease of >3/min in the RR was a risk for respiratory depression. As the effect of suppositories or orally administered drugs may require a certain duration for onset, the lowest RR observed in the following 6-hour period was determined. The lowest RR was observed in 14 patients (58%) during the first hour after the administration of benzodiazepines. In contrast, the lowest RR was observed in the remaining 10 patients, 2–6 hours after the administration of benzodiazepines. Three of these patients received a rescue dose of opioids after the administration of benzodiazepines; no relation between the opioid dose and timing of RR decrease was observed in the remaining patients.

Assessment of the lowest RR revealed that eight patients matched the criteria for respiratory depression, that is, a decrease of >3/min in RR. These data were compared with the RR changes observed at the same time on the previous day. Among the 18 patients with available records for the previous day, six patients showed a decrease of >3/min. On average, the RR changed from 12.0  ±  3.9/min to 10.3  ±  3.3/min (*n* = 24, *p* = 0.0005) after the administration of benzodiazepines. This change did not differ from the decrease in RR observed on the previous day (12.1  ±  3.3/min to 10.3  ±  3.4/min, intergroup difference: *p* > 0.83). All other respiratory variables were compared between T0 and T, when the RR was at its lowest. Comparison of the apnea–hypopnea frequency, RRSI, TW, and TWSI at T0 and T, when the RR was at its lowest, revealed no significant differences (Table 2). Additional comparison was performed between patients with RR reduction of ≥3/min and <3/min (Table 2). Eight patients with RR reduction by ≥3/min were observed with a trend but insignificant increase in apnea–hypopnea frequency (*p* = 0.075). However, it is noteworthy that one patient was observed with an increase by 24/min of apnea–hypopnea frequency.

**Table 2.** Comparison of Respiratory Variables Between T = 0 and T at Lowest RR, on the Day of Benzodiazepine Administration

	Total (n = 24)			RR reduction by ≥3/min (n = 8)			RR reduction by <3/min (n = 16)			
	T = 0	At lowest RR	p	T = 0	At lowest RR	p	T = 0	At lowest RR	p	Intergroup difference
Respiratory rate (/min)	11 (7, 23)	9 (6, 21)	<0.001	15 (10–23)	12 (6–16)	0.008	10 (7–20)	9 (7–21)	0.034	
RR change				−3.5 (−7 to −3)			−0.5 (−2 to 1)			<0.001
Apnea-hypopnea frequency	0 (0, 47)	1 (0, 43)	0.37	0 (0–7)	3.5 (0–31)	0.09	0.5 (0–47)	1 (0–43)	0.65	
Apnea–hypopnea frequency change				4 (−2 to 24)			0 (−6 to 5)			0.075
RRSI (%)	16.8 (5.6, 52.6)	14.4 (3.7, 53.3)	0.09	22.5 (9.4–52.6)	15.6 (7.9–53.3)	0.49	12.7 (5.6–34.1)	14.4 (3.7–25)	0.07	
TW (g)	59.7 (29.4, 171)	62.2 (30.1, 187)	0.54	67.5 (44.3–123)	73.5 (43.5–154)	0.41	56.7 (29.4–171)	61.4 (30.1–187)	0.35	
TWSI (%)	10.5 (4.9, 32.7)	10.3 (5.0, 57.8)	0.79	15.0 (6.1–32.7)	10.5 (8.2–57.8)	0.86	10.4 (4.9–20.3)	10.3 (5.0–22.7)	0.62	

Values are presented as median (min–max).

RR, respiratory rate; RRSI, respiratory rate stability index; TW, tidal weight; TWSI, tidal weight stability index.

**FIG. 3.**
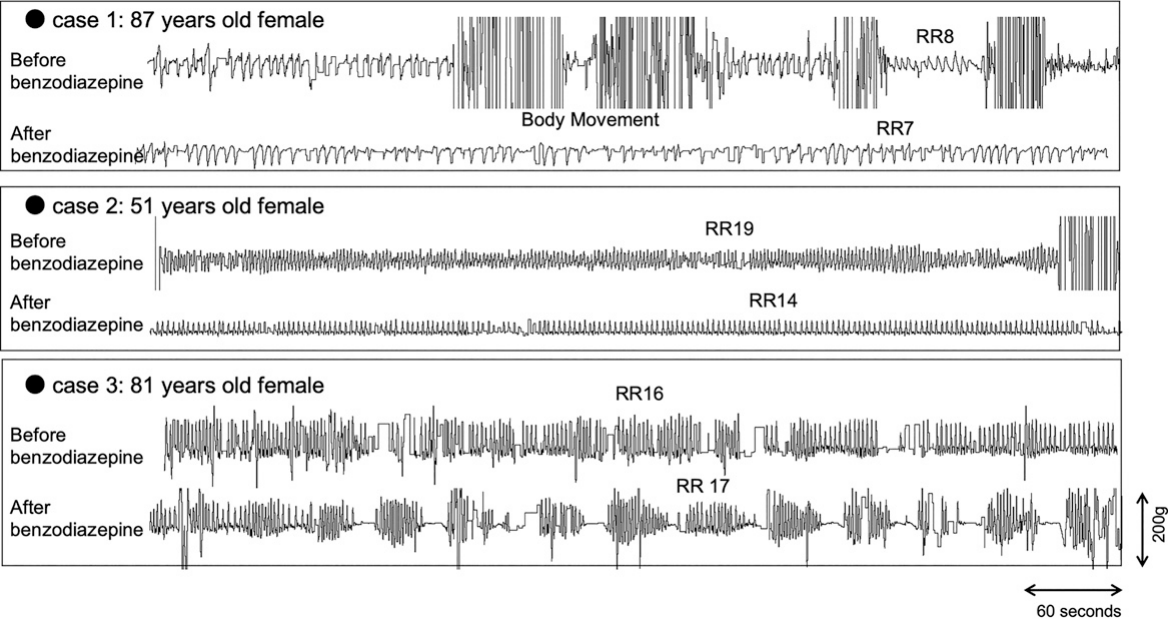
Typical traces of respiration from the BSS sensors. Case 1: Lung cancer, chief complaint: dyspnea, daytime, oral hydromorphone 6 mg/day. The body movement ceased and respiration became constant after the rectal administration of 3 mg of bromazepam. Case 2: Breast cancer, chief complaint: insomnia, nighttime, oral oxycodone 10 mg/day. The respiratory rate decreased by 5/min after the rectal administration of 4 mg of diazepam; however, the tidal volume and rate intervals were constant after the administration of benzodiazepine. No relevant respiratory depression was observed. Case 3: ovarian cancer, chief complaint: stomach distention, nighttime, transdermal fentanyl 0.5 mg/day. The oral administration of 1 mg of diazepam was effective in alleviating some of her symptoms. The respiratory pattern changed after the administration of benzodiazepine; however, periodic breathing was also observed on the previous night, indicating that the administration of benzodiazepine did not induce a new episode. BSS, Bed Sensor System.

Figure 3 shows typical traces of respiration from the BSS sensors. Case 1 was a female patient with lung cancer who presented with dyspnea. Frequent body movement was observed in this patient owing to inability to rest. The body movement ceased, and respiration became constant following rectal administration of 3 mg of bromazepam. Case 2 received rectal administration of 4 mg of diazepam for the management of insomnia. The RR decreased by 5/min; however, the tidal volume and rate intervals remained constant after the administration of benzodiazepine, and no relevant respiratory depression was observed. Case 3 was a patient with stomach distention owing to ascites caused by ovarian cancer. Oral administration of diazepam was effective in alleviating some of the symptoms. The respiratory pattern changed following the administration of benzodiazepine; however, periodic breathing was also observed the preceding night, indicating that the administration of benzodiazepines did not induce any new episodes.

### Effect of repeated administration of benzodiazepines

Of the 24 patients observed, 9 were evaluated for the effects of repeated benzodiazepine dosing. The results of five repeated doses are summarized in Table 3. A decrease in the RR was consistently observed from the first dose, with no patient experiencing an RR of less than 8/min. The median dosing interval was 24 hours; however, benzodiazepines were administered within 10 hours (ranging from 2–9 hours) on five occasions, with RR reductions varying from 0 to 7 breaths/min. There was no significant association between the administration intervals and changes in RR (*r* = −0.025, *p* = 0.883). Similarly, no significant deterioration was observed in the RRSI, TW, and TWSI. Repeated benzodiazepine doses for symptom relief in patients receiving continuous opioids showed no cumulative effect.

**Table 3.** Respiratory Parameters After Repeated Benzodiazepine Dosing (*n* = 9)

	1st	2nd	3rd	4th	5th	p-Value
Benzodiazepines dose (mg)	6 (1–6)	6 (1–10)	6 (1–6)	6 (1–10)	6 (1–10)	0.199
Administration interval (hours)	N/A	24 (5–211)	23 (2–24)	23 (3–73)	24 (7–60)	0.89
ACI at T0	130 (44–541)	122 (68–296)	162 (18–384)	177 (32–305)	178 (22–406)	0.885
Delta-ACI (T0—lowest RR)	69 (−30 to 406)	69 (−219 to 199)	101 (−189 to 284)	73 (−19 to 163)	102 (−335 to 242)	0.9
RR at T0 (/min)	10 (8–20)	14 (10–22)	12 (9–17)	13 (9–17)	13 (9–20)	0.419
Lowest RR (/min)	11 (8–21)	12 (8–21)	11 (9–17)	12 (9–18)	13 (9–19)	0.493
Delta-RR (T0—lowest RR) (/min)	0 (−1 to 3)	1 (1–5)	2 (−1 to 4)	1 (−1 to 4)	1 (−4 to 7)	0.331
RRSI at T0 (%)	9.8 (5.6–22.0)	10.7 (4.0–22.0)	13.5 (5.6–20.6)	9.4 (4.8–72.9)	12.8 (4.8–22.2)	0.838
Delta-RRSI (T0—lowest RR) (%)	0 (−1.5 to 11.7)	0.2 (−4.6 to 8.8)	1.3 (−3.5 to 8.9)	0.3 (−1.0 to 45.5)	−0.1 (−50.4 to 15.1)	0.84
TW at T0 (g)	55.9 (29.5–147)	59.8 (12.9–134)	58.6 (19.7–122)	59.6 (14.4–114)	66.4 (24.4–112)	0.584
Delta-TW (T0—lowest TW) (g)	−3.3 (−46.3 to 56.3)	−9.5 (−18.0 to 5.5)	−2.9 (−25.0 to 14.6)	−4.9 (−21.5 to 4.0)	0.9 (−42.2 to 39.0)	0.95
TWSI at T0 (%)	10.1 (7.0–19.2)	9.6 (5.7–23.4)	9.9 (5.2–16.6)	12.2 (6.2–44.6)	11.8 (6.0–23.4)	0.431
Delta-TWSI (T0—lowest RR) (%)	0.5 (−6.3 to 5.6)	0.1 (−6.5 to 9.3)	−1.8 (−11.3 to 3.2)	3.0 (−3.1 to 31.0)	0.3 (−33.8 to 13.8)	0.328

ACI, activity index; RR, respiratory rate; RRSI, respiratory rate stability index; TW, tidal weight; TWSI, tidal weight stability index.

## Discussion

The administration of a clinical dose of benzodiazepine to patients with end-stage cancer under opioid analgesia did not add the risk of respiratory depression already relevant with opioid analgesics in this prospective observational study.

### Monitoring of respiratory depression

Benzodiazepines have shown great potential for relieving some serious symptoms experienced by patients receiving palliative care.^16^ However, they are not dispensed by some clinicians owing to the fear of respiratory depression. Opioids alone, which are most often prescribed to patients with terminal cancer, can also have respiratory depressive effects; however, the side effects are within the clinically acceptable range when an appropriate dose is used to alleviate the symptoms of pain and dyspnea.^3^ The coadministration of opioids and benzodiazepines may synergistically enhance respiratory depressant effects via their combined effect on the medullary respiratory center.^17^ The likelihood of the incidence of in-hospital cardiac arrest increases substantially when both drugs are used together.^18^ Furthermore, benzodiazepines may be involved in inpatient deaths during the so-called opioid crises.^19^ Thus, it is important to determine whether patients receiving palliative care experience severe respiratory depression due to the combination of clinical dose of opioids and benzodiazepines. However, continuous monitoring of respiration in terminally ill patients was not feasible until recently, as normal monitoring limits patient activity and imposes a burden on the patient, thereby acting as a major limitation to long-term continuous research, including nighttime.

A noncontact, nonrestraining vital sign monitor that facilitates continuous and detailed measurement of respiration, sleep time, and activity patterns in bed was used in this study. This was the first study to continuously monitor the direct effect of benzodiazepines on respiration for up to 6 hours, as well as their repeated effects in critically ill patients.

### Benzodiazepine and respiratory depression

In this study, a decrease of >3/min in the RR was observed in the 6 hours after the administration of benzodiazepine. The lowest RR was observed within 1 hour of the administration of benzodiazepine in 58% of patients and within 2–6 hours of the administration of benzodiazepine in the remaining 42% of patients. The decrease in RR can be considered respiratory depression, as well as a result of symptom alleviation and sleep. Patients receiving opioids for analgesia are prone to have decreased RR, especially during sleep. This was also apparent in this study, wherein the decrease in RR was similar to that observed on the previous day with only opioids. Clemens et al. reported the efficacy and safety of combination therapy of opioids and benzodiazepines using transcutaneous CO_2_ and SpO_2_ monitors in their short-term observational study; however, the monitoring was performed only during daytime in their study.^3^ Notably, even continuous SpO_2_ monitoring, which is considered noninvasive in the field of medicine, can be difficult in patients receiving palliative care when performed continuously for 24 hours. The present study clearly demonstrated that the RR decreased after the administration of benzodiazepines to a similar level observed with opioid and natural sleep. The size of respiration, speculated by the TW, and regularity of respiration, assessed using RRSI, TWSI, and apnea–hypopnea frequency, were unaffected by the administration of benzodiazepines. This effect is noticeably different from that of opioids, the administration of which decreases the RR and increases respiratory instability, resulting in ataxic breathing.^5^

### Limitations

This study had some limitations that hinder the generalization of the findings. First, the small sample size does not preclude the possibility of relevant respiratory depression occurring in patients following coadministration of benzodiazepines and opioids. Second, whether the respiratory changes observed in this study can be attributed to the administration of benzodiazepines is difficult to conclude owing to the lack of control for patients who only received opioids. However, conducting a randomized study on patients with terminal illnesses by withholding treatments that are clinically effective in relieving their symptoms is ethically challenging. Therefore, the precise monitoring of respiration before and after drug administration remains a significant challenge in this field.

The effect of repeated benzodiazepine doses could only be assessed in a small number of patients (*n* = 9). Respiration, an important vital sign in the care of terminally ill patients, can change dramatically as the terminal stage progresses. Therefore, only patients with a similar length of life could be used for repeated dose analysis. Furthermore, the dose-dependent effect must be addressed in future studies.

## Conclusions

The predicted decrease in RR following the nonintravenous administration of benzodiazepine for alleviating symptoms was observed in one-third of patients receiving opioids in palliative care, which was similar to the event observed on the previous day without the administration of benzodiazepines. Notably, the decrease in RR was not accompanied by a reduction in respiratory size or irregularities that could predict serious deterioration in respiration. As individual changes are still not predictable, continuous monitoring without patient burden may be of crucial importance.
